# Malaria epidemiology in an area of stable transmission in tribal population of Jharkhand, India

**DOI:** 10.1186/s12936-017-1833-9

**Published:** 2017-05-02

**Authors:** Manoj K. Das, Brijesh K. Prajapati, Régis W. Tiendrebeogo, Kumud Ranjan, Bright Adu, Amit Srivastava, Harvinder K. Khera, Narendra Chauhan, Sanjay Tevatiya, Ikhlaq H. Kana, Surya Kant Sharma, Subhash Singh, Michael Theisen

**Affiliations:** 1 Field Unit, National Institute of Malaria Research, Ranchi, Jharkhand India; 2 National Institute of Malaria Research, Indian Council of Medical Research, New Delhi, 110077 India; 3 Department for Congenital Disorders, Statens Serum Institut, Artillerivej 5, 2300 Copenhagen, Denmark; 4 Centre for Medical Parasitology at Department of International Health, Immunology and Microbiology, University of Copenhagen, Copenhagen, Denmark; 5 Department of Infectious Diseases, Copenhagen University Hospital, Rigshospitalet, Copenhagen, Denmark; 6 Noguchi Memorial Institute for Medical Research, University of Ghana, Legon, Ghana; 7 Indian Institute of Integrative Medicine, Canal Road, Jammu, 180001 India

**Keywords:** Malaria, *Plasmodium falciparum*, *Plasmodium vivax*, Morbidity, Age, Jharkhand, India

## Abstract

**Background:**

Malaria remains an important health problem in India with approximately 1 million cases in 2014. Of these, 7% occurred in the Jharkhand state mainly in the tribal population.

**Methods:**

This study was conducted in Dumargarhi, a tribal village about 42 km east of Ranchi city, Jharkhand, from May 2014 to September 2016. Four point prevalence surveys were carried out during consecutive high (October–December) and low (June–August) transmission seasons. Malaria cases were recorded from April 2015 to April 2016 through fortnightly visits to the village. Adult mosquito densities were monitored fortnightly by manual catching using suction tube method.

**Results:**

The study area consists of five hamlets inhabited by 945 individuals living in 164 households as recorded through a house-to-house census survey performed at enrollment. The study population consisted predominantly of the Munda (n = 425, 45%) and Oraon (n = 217, 23%) ethnic groups. Study participants were categorized as per their age 0–5, 6–10, 11–15 and >15 years. There were 99 cases of clinical malaria from April 2015 to April 2016 and all malaria cases confirmed by microscopy were attributed to *Plasmodium falciparum* (94 cases) and *Plasmodium vivax* (5 cases), respectively. During the high transmission season the mean density of *P. falciparum* parasitaemia per age group increased to a peak level of 23,601 parasites/μl in the 6–10 years age group and gradually declined in the adult population. Malaria attack rates, parasite prevalence and density levels in the study population showed a gradual decrease with increasing age. This finding is consistent with the phenomenon of naturally acquired immunity against malaria. Three vector species were detected: *Anopheles fluviatilis, Anopheles annularis,* and *Anopheles culicifacies.* The incoherence or complete out of phase pattern of the vector density peaks together with a high prevalence of parasite positive individuals in the study population explains the year-round malaria transmission in the study region.

**Conclusions:**

The collection of clinical data from a well-characterized tribal cohort from Jharkhand, India, has provided evidence for naturally acquired immunity against malaria in this hyperendemic region. The study also suggests that enforcement of existing control programmes can reduce the malaria burden further.

## Background

Malaria is a vector borne disease caused by five different *Plasmodium* species. Of these, *Plasmodium falciparum* and *Plasmodium vivax* are the main causes of disease and mortality worldwide [1]. Today, malaria is primarily confined to the poorest tropical areas of Africa, Asia and Latin America, where it contributes as one of the world’s greatest public health problems.

In India, about 1.0 million malaria cases were reported in 2014 of which approximately 7% originate from Jharkhand state (National Vector Borne Disease Control Programme of the Government of India). The forest, hilly terrain, favorable climate, inaccessible area, tribal culture, migration and social unrest are the main contributing factors to the malaria burden in Jharkhand [2]. An estimated 54 million tribals belonging to about 40 ethnic communities (constituting around 28% of the total population of Jharkhand) reside in the forest areas. This tribal population accounts for 8% of the total population of India but contributes to 30% of all malaria cases [3]. Introduction of new epidemiological tools, bivalent rapid diagnostic test (RDT) kits for diagnosis, artemisinin-based combination therapy (ACT) for the treatment of *P. falciparum* malaria and long lasting insecticidal nets (LLINs) has reduced the annual parasite incidence (API) of Jharkhand state from 6.20 in 2010 to 2.55 in 2015 (MK Das and SK Sharma, unpublished). Despite these improvements, malaria remains a major public health problem causing morbidity and mortality in this region.

Individuals who are naturally exposed to malaria develop a strong strain-transcending immunity [4, 5] which takes years of exposure to develop and is characterized by a low grade parasitaemia in the presence of vigorous *P. falciparum*-specific immune responses [6]. This immunity, termed premunition, has been extensively characterized in Africa [7–10] primarily because this is where the heaviest malaria related morbidity and mortality occurs [1]. However, with the current focus on malaria eradication, it has become more crucial than ever to investigate malaria epidemiology in other parts of the world. Few such studies have been performed on the Indian sub-continent where malaria transmission rates vary considerably over short geographical distances [11, 12].

The present study describes the demographic profile and provides an overview of the malaria transmission dynamics in a malaria endemic tribal population in Jharkhand, India.

## Methods

### Study design, area, and population

**Fig. 1:**
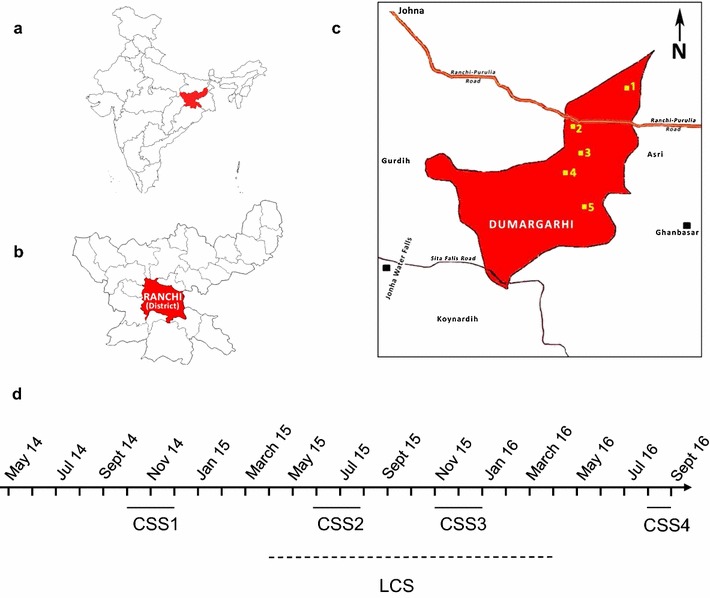
Map of the study region showing locations of **a** Jharkhand state in the Union of India. **b** Ranchi District. **c** Study site in Ranchi district including the hamlets Karam Tungri (*1*), Jarawadih (*2*), New Torang (*3*), Old Torang (*4*), and Dumargarhi (*5*). **d** Timing and duration of cross sectional (CSS) and longitudinal surveys (LCS) is shown

This study was conducted in Dumargarhi village (Ranchi district; Fig. 1; Table 1) about 42 km east of Ranchi city, Jharkhand from May 2014 to September 2016. The study included four cross sectional surveys (CSS) to determine the point prevalence of *P. falciparum* and *P. vivax* during high and low transmission periods, respectively (Fig. 1d). A longitudinal cohort survey (LCS) was conducted between April 2015 and April 2016 to determine the burden of clinical malaria (Fig. 1d). The study area is situated in a forested area with terrain full of high and lowlands and a tropical humid climate. It receives high rainfall during Southwest monsoon (mid-May–September) and low rainfall during Northeast monsoon (December–February) with an average annual rainfall of 153 cm. The weather seasons are hot dry summer from April to mid-May, a monsoon from mid-May to September, autumn from October to November, winter from December to January, and spring from February to March. The maximum temperature during summer rises to around 41 °C and the minimum temperature during winter falls to about 5 °C. The relative humidity ranges between 35 and 90%. Malaria transmission occurs year round but peaks after the monsoon season from October to December. The study village consists of five hamlets located 1–3 km from each other; these hamlets are connected by an all-weather road. The nearest primary health center (PHC) is located 20 km from the study site. The study population belonged to different ethnic groups consisting of the Munda and the Oraon (the major ethnic groups) and the other remaining ethnic groups were the Lohra, Bedia, Baraik and Kachhap. Each ethnic community has its own socio-cultural traditions but they live in harmony with other groups. Inter marriages between different ethnic communities are rare and not solicited by the community leaders. Most villagers work as subsistence farmers, but some are engaged as daily wage laborers. Village children attend a local primary school where they get free mid-day meal under the Central Government sponsored scheme to prevent malnutrition among school going children and also as an incentive to prevent school drop outs.

**Table 1:** Demographic and baseline characteristics

Factor	Dumargarhi	Jarwadih	New Torang	Karam Tungri	Old Torang	Total	*P* value*
Number of individuals (%)	186 (19.7)	103 (10.9)	84 (8.9)	123 (13.0)	449 (47.5)	945 (100.0)	
Age group (years)							
0–5	14 (1.5)	9 (0.9)	9 (0.9)	9 (0.9)	71 (7.5)	112 (11.9)	
6–10	30 (3.2)	11 (1.2)	5 (0.5)	18 (1.9)	45 (4.8)	109 (11.5)	
11–15	19 (2.0)	9 (1.0)	8 (0.8)	19 (2.0)	33 (3.5)	88 (9.3)	
≥16	123 (13.0)	74 (7.8)	62 (6.6)	77 (8.1)	300 (31.7)	636 (67.3)	*0.001*
Sex							
Female	90 (9.5)	49 (5.2)	42 (4.4)	56 (5.9)	215 (22.8)	452 (47.8)	
Male	96 (10.2)	54 (5.7)	42 (4.4)	67 (7.1)	234 (24.8)	493 (52.2)	0.99
Education							
G	1 (0.1)	0 (0.0)	0 (0.0)	1 (0.1)	0 (0.0)	2 (0.2)	
H	15 (1.8)	7 (0.9)	26 (3.2)	33 (4.1)	43 (5.3)	124 (15.3)	
I	82 (10.1)	31 (3.8)	22 (2.7)	30 (3.7)	204 (25.1)	369 (45.4)	
Mi	31 (3.8)	4 (0.5)	22 (2.7)	29 (3.6)	48 (5.9)	134 (16.5)	
Pr	33 (4.1)	13 (1.6)	6 (0.7)	18 (2.2)	74 (9.1)	144 (17.7)	
U	12 (1.5)	1 (0.1)	0 (0.0)	0 (0.0)	26 (3.2)	39 (4.8)	*<0.005*
Profession							
A	33 (20.5)	16 (9.9)	13 (8.1)	20 (12.4)	77 (47.8)	159 (98.8)	
NIL	0 (0.0)	0 (0.0)	1 (0.6)	0 (0.0)	0 (0.0)	1 (0.6)	
S	1 (0.6)	0 (0.0)	0 (0.0)	0 (0.0)	0 (0.0)	1 (0.6)	0.159
Income							
[0–50,000]	25 (15.3)	20 (12.3)	13 (8.0)	20 (12.3)	71 (43.6)	149 (91.4)	
[50,000–100,000]	8 (4.9)	0 (0.0)	0 (0.0)	0 (0.0)	6 (3.7)	14 (8.6)	*0.002*
Housing							
M	182 (20.1)	100 (11.0)	34 (3.7)	113 (12.5)	431 (47.5)	860 (94.8)	*<0.005*
P	0 (0.0)	0 (0.0)	8 (0.9)	0 (0.0)	0 (0.0)	8 (0.9)	
T	0 (0.0)	0 (0.0)	39 (4.3)	0 (0.0)	0 (0.0)	39 (4.3)	
Bednet							
N	142 (15.7)	100 (11.0)	81 (8.9)	113 (12.5)	367 (40.5)	803 (88.5)	
Y	40 (4.4)	0 (0.0)	0 (0.0)	0 (0.0)	64 (7.1)	104 (11.5)	*<0.005*

Education: *G* graduate, *H* high school education (intermediate), *I* illiterate, *Mi* middle, *Pr* primary, *U* undergraduate; Profession: *A* agriculture, *NIL* no profession, *S* service; Housing: *M* mud walls with thatched roof, *P* cement walls and roof, *T* brick walls with tin roof, Bednet: *Y* yes; *N* no

* *P* value is based on Chi Square test

### Longitudinal cohort surveys (LCS)

Malaria cases were recorded from April 2015 to April 2016 in a longitudinal cohort study. An episode of clinical malaria was defined as fever (auxiliary temperature ≥36.5 °C, measured or reported) with slide positive for any asexual *P. falciparum* and/or *P. vivax* parasitaemia and/or at least one other sign of malaria such as vomiting, diarrhoea, or malaise. A trained field worker visited every house in all hamlets once in a fortnight on a fixed schedule for active surveillance which involved recording auxiliary temperature and all febrile individuals were examined using a rapid diagnostic test (RDT) kit [SD Bioline Malaria Ag *Pf*/HRP-2/*p*LDH, 3 band kit Alere Inc.]. RDT positive cases were examined by thick and thin blood smears from finger prick blood samples. All slides collected from the study area were brought to a laboratory at the National Institute of Malaria Research field unit, Itki, Ranchi. Slides were examined by trained microscopists under compound microscope at 100× magnification after staining with Jaswant Singh Bhattacharjee (JSB) stain (Rankem, India). Asexual parasites were counted against 200 leukocytes and parasite density was calculated as number of asexual parasites per micro liter of blood assuming a mean normal leukocyte count of 8000/µL. Slide-positive cases were provided anti-malarial treatment as per the guidelines of the National Vector Borne Disease Control Programme of the Government of India [13, 14]. A blood smear was classified negative if no parasite was found in 100 random microscopic fields. In between the fortnightly visits, passive surveillance of malaria cases was maintained through telephonic reporting of any febrile cases to the trained field worker by family members of the patient. Confirmation of these reported cases was done by the trained field worker through use of RDT kit and malaria positive cases were treated as described above. Presumptive treatment was not given to the patients.

### Cross-sectional surveys (CSS)

Four point prevalence surveys were carried out in all hamlets twice during peak transmission (October–December) and twice during low malaria transmission seasons (June–August). All villagers who were available and willing to participate were included in these surveys. The study subjects were examined for malaria by RDT kit and microscopy of blood smears was performed irrespective of clinical symptoms. Malaria cases were treated as per the national drug policy described above. Common ailments were treated by the health-care workers, whereas individuals with serious life threatening diseases such as TB, hypertension, respiratory and liver disorders were referred to Angara primary health center or to a referral hospital located in Ranchi city. The relevant information for each study subject such as name, age, sex, resident status, fever, auxiliary temperature, and other clinical symptoms were recorded in a patient data sheet and a computerized data base for such information was developed.

### Vector species and entomological surveys

The district is characterized by a large network of streams and other water bodies which provide innumerable and diverse breeding sites for species-specific malaria vectors [*Anopheles culicifacies, Anopheles fluviatilis* and *Anopheles annularis* (Diptera: Culicidae)] throughout the year. *An. culicifacies* breeds mainly in pools formed in streams and riverbeds; the most productive breeding sites of *An. fluviatilis* are slow-moving streams while *An. annularis* breeds in margins of ponds, rivers and streams with abundant vegetation. The three vector species *An. culicifacies* (sibling species B & C), *An. fluviatilis* (sibling species T) and *An. annularis* (sibling species A) were identified based on their morphological characteristics and cytogentic analysis of their polytene chromosomes [15].

Entomological survey was conducted fortnightly from May 2014 to March 2016 by manual catching using suction tube method [16, 17]. Indoor resting collections were made in the morning between 06:00 and 08:00 h from four randomly selected human dwellings and four cattle sheds. Mosquitoes from each dwelling were kept separately in test tubes and species were identified based on morphology [18–20]. The sibling species identity of the captured vector species was determined through cytogenetic analysis of the polytene chromosomes [15]. Densities per person-hour searching (person-hour density or PHD) of total anophelines and vector species were determined.

### Statistical analysis

Statistical analysis was done with the R 3.3.0 and GraphPad Prism 5 software. Age was categorized in four levels (0–5, 6–10, 11–15 and >15 years) and demographic characteristics were compared between villages by using the χ^2^-test. *P* values <0.05 were considered to indicate statistical significance.

## Results

### Population characteristics

The study area consists of five hamlets inhabited by 945 individuals living in 164 households as recorded through a house-to-house census performed at enrollment. The population distribution within these hamlets were Dumargarhi (n = 186), Jarwadih (n = 103), New Torang (n = 84), Karam Tungri (n = 123) and Old Torang (n = 449) (Table 1). The mean age of the study population was 27.5 ± 18.8 years. Villagers were divided into 4 age groups: ≤5 years (n = 112, 11.9%), 6–10 years (n = 109, 11.5%), 11–15 years (n = 88, 9.3%) and >15 years (n = 636, 67.3%). The study population consisted predominantly of the Munda (n = 425, 45%) and Oraon (n = 217, 23%) ethnic groups. The remaining villagers belonged to Lohra, Bedia, Baraik and Kachhap ethnic groups. Individuals who used bed net constituted 11.5% of the population. The gender distribution was not significantly different between villages (P < 0.99) while the distribution of age, bed net use, housing, income was significantly different between villages (P < 0.005, χ^2^ analysis) (Table 1).

### *Plasmodium falciparum* infections recorded through fortnightly surveillance

**Fig. 2:**
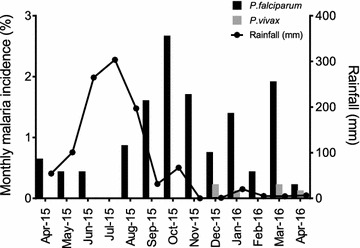
Monthly malaria incidence (cases per 945 population) in the Ranchi study cohort from April 2015 to April 2016. During this period there were 94 *P. falciparum* and 5 *P. vivax* cases diagnosed with febrile malaria. Monthly incidence of *P. falciparum* (*black bars*)*, P. vivax* (*grey bars*) is shown. The *line plot* is the monthly rainfall recorded during the period

**Fig. 3:**
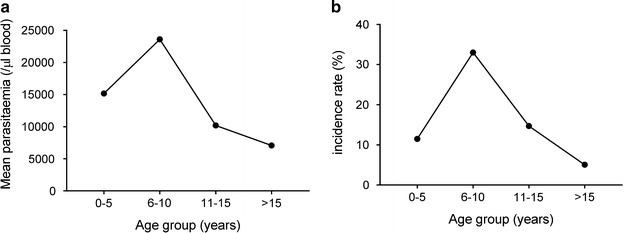
Mean *P*. *falciparum* density and febrile malaria incidence per age-group. **a** Mean parasite density of all slide positive *P. falciparum* malaria cases recorded during the longitudinal follow-up from April 2015 to April 2016 was calculated per age groups. **b** Incidence rate of febrile malaria in the same age groups

Malaria transmission was perennial and *P. falciparum* malaria was reported throughout the year (Fig. 2). There were 99 cases of clinical malaria from April 2015 to April 2016 and all cases confirmed by microscopy were attributed to *P. falciparum* (94 cases) and *P. vivax* (5 cases), respectively. The monthly incidence of *P. falciparum* and *P. vivax* is shown in Fig. 2. *P. falciparum* malaria incidence was generally higher after the end of the monsoon season and lower in the hot dry summer months. The mean density of *P. falciparum* parasitaemia was calculated for the 0–5, 6–10, 11–15, and >15 year age groups, respectively (Fig. 3a). It increased to a peak level of 23,601 parasites/μl in the 6–10 years age group and gradually declined in the adult population to a level of 7066 parasites/μl. A similar pattern was observed in the incidence rate of febrile *P. falciparum* malaria (Fig. 3b).

### Malaria parasite prevalence during high and low transmission periods

During the high transmission season (CSS1 and CSS3), 386 and 327 villagers respectively were examined for *P. falciparum* and *P. vivax* infections by RDT kit (Table 2). Of these, 180 were positive for malaria parasites (*Pf* = 148, *Pv* = 10, mixed infection = 22) in CSS1 and 72 were positive for malaria (*Pf* = 59, *Pv* = 9, mixed infection = 4) in CSS3. The parasite rate during the first and second surveys in the high transmission was 46.6 and 22% respectively. During low transmission (CSS2 and CSS4), 254 and 225 individuals were enrolled, 50% of whom had also participated in CSS1. Of these, 51 individuals were malaria positive by the RDT test in CSS2 (*Pf* = 46, *Pv* = 2, mixed infections = 3) and 39 were positive for malaria in CSS4 (*Pf* = 35, *Pv* = 4). The parasite rate during the two surveys in the low transmission was 20.1 and 17.3% respectively. The average parasite rate during the high (CSS1 + CSS3) and low (CSS2 + CSS4) transmission season was 35.3 and 18.8%, respectively. A significant variation in the seasonal malaria prevalence (P < 0.0007, χ^2^ analysis) was observed.

**Table 2:** Malaria prevalence and mean parasite density during high and low transmission seasons in the study population

Transmission season	Study	RDT performed	Total positive	*Pv*	*Pf*	Mix	*Pf* parasite rate (%)	*Pv* parasite rate (%)	Mean *Pf* parasite density^a^	*Pf* malaria prevalence (%)
High (Oct–Dec 2014)	CSS1	386	180	10	148	22	44	8.3	6113 (±1456)	11.4
Low (Jun–Aug 2015)	CSS2	254	51	2	46	3	19.3	1.9	3480 (±1511)	2.8
High (Oct–Dec 2015)	CSS3	327	72	9	59	4	19.3	3.9	17,195 (±7854)	8.6
Low (Jun–Aug 2016)	CSS4	225	39	4	35	0	15.6	1.8	1689 (±2483)	0.0

*RDT* Rapid diagnostic test. *Pf Plasmodium falciparum*, *Pv Plasmodium vivax*. Mix; *Pf* and *Pv*

^a^Parasitemia was determined by microscopy

**Fig. 4:**
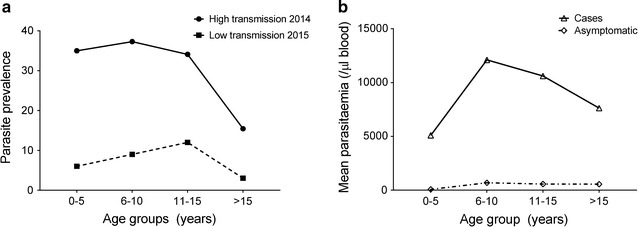
**a** Age-dependent prevalence of *P. falciparum* positive individuals during the high (*circles*) and the low (*squares*) transmission seasons. **b** Mean *P. falciparum* density per age group in acute malaria cases (*triangles*) and asymptomatic carriers (*diamonds*) during high transmission season in 2014 (CSS1). Parasite densities of acute malaria cases were approximately 66-fold higher than those of asymptomatic carriers in the 0–5 years age group and this threshold decreased with age

Eighty-six (47.8%) and 13 (25.5%) of the RDT positive samples were also found to be slide positive for *P. falciparum* from CSS1 and CSS2, respectively. The mean *P. falciparum* parasite density of these slide positive individuals was 6113 (±1456) parasites/μl and 3480 (±1511) parasites/μl during high and low transmission season, respectively (Table 2). The prevalence of *P. falciparum* parasitaemia was highest in children up to 15 years of age and declined in the adult population during both high and low-transmission season (Fig. 4a). During CSS1, there were 44 cases of *P. falciparum* malaria. The remaining slide positive individuals (n = 42) who did not have fever or any other symptom of malaria were classified as asymptomatic *P. falciparum* carriers. The number of malaria cases and asymptomatic carriers were 4, 15, 10, and 15 and 3, 7, 5, and 27 in the 0–5, 6–10, 11–15, and >15 years age groups, respectively. The mean parasite density of each age group was plotted for malaria cases and asymptomatic carriers, respectively (Fig. 4b). Parasite densities of acute malaria cases were 66-fold higher in the 0–5 age group than those of asymptomatic carriers (Fig. 4b). During CSS3 and CSS4 lower prevalence of malaria observed in this study population could be attributed to the ongoing case surveillance during the preceding study period and effective treatment of the malaria positive cases, thereby reducing the parasite transmission.

### Malaria vectors and seasonal prevalence

**Fig. 5:**
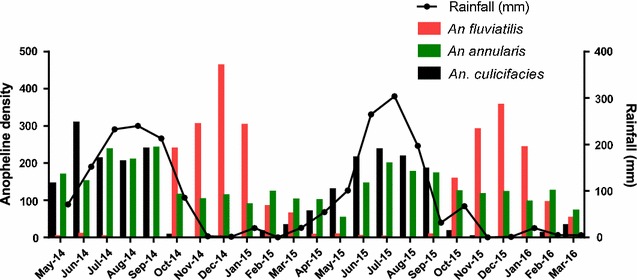
Monthly density of *An. culicifacies* (*black bars*) *An. Annularis* (*green bars*), and *An. fluviatilis* (*red bars*) in the study area based on indoor resting hand catch collections once every month. The *line plot* is the monthly rainfall recorded during the period

Average annual vector density determination in the study area established *An. fluviatilis* as the most prevalent vector constituting 49% of all the vector species. *An. annularis* and *An. culicifacies* constituted 34 and 17%, respectively. The cumulative annual average of all the vectors captured in the study area during fortnightly surveys was 21 and 79% for vectors resting in human dwellings and cattle sheds, respectively. Some seasonal fluctuations in the month-wise person-hour density (PHD) of *An. culicifacies, An. fluviatilis* and *An. annularis* was observed (Fig. 5). The highest density of *An. fluviatilis* was observed between October and February, whereas the highest density of *An. culicifacies* and *An. annularis* was observed between May and September and the lowest during October–April.

## Discussion

Malaria is a focal disease in India, influenced by several local ecological and social factors [21]. The study team collected samples and clinical data from 945 individuals of tribal origin living in closely placed hamlets in the forested hills of Jharkhand in the Eastern parts of India. This study involved four cross-sectional surveys two each during the high (October–December) and the low (June–August) transmission seasons and fortnightly surveillance during the periods intervening the cross-sectional surveys. In summary: (1) malaria transmission was hyper-endemic, and (2) there was evidence suggesting that the population develops naturally acquired immunity (NAI) as a function of age and exposure. The observations that children had (i) a high incidence and prevalence rate of *P. falciparum* infections, (ii) a high malarial attack rate, and (iii) higher parasite densities compared to adults are indications of hyper-endemicity in this study area. It is generally observed that individuals living in hyper-endemic areas gradually develop an anti-parasite immunity which protects them from febrile malaria. The age-dependent variation of malaria attack rates, parasite prevalence and density levels in the present study was characterized by a gradual decrease with increasing age. This relationship is typical of NAI against malaria and is similar to findings in highly endemic areas of Africa [22–24] and Asia [25]. The development of NAI is further underscored by the very high difference in parasite densities between acute malaria cases and asymptomatic carriers. The authors observed that parasite densities in acute malaria cases were much higher than those in asymptomatic carriers. This difference was particularly high in young children and decreased with age i.e. the parasite density causing fever decreases with age and exposure. This finding is related to the phenomenon of the pyrogenic threshold which has been described in Africa [26]. Similar age-dependent decrease in the prevalence of parasitaemia and febrile malaria has also been observed in Odisha, which is located in a neighboring state of India [27]. Another study in the Sundargarh district in Odisha, also demonstrated high levels of agglutinating antibodies against *P. falciparum* infected erythrocytes in healthy adults but not in children below 3 years of age [28]. Collectively these findings support the notion that individuals who live in highly malaria endemic areas of India develop NAI.

Another important observation of the present study was the apparent decrease in the malaria parasite burden between 2014 and 2016. In the first cross-sectional survey during high transmission season of 2014 the rate of *P. falciparum*-positive samples was around 44% with a clinical malaria prevalence of 11.4%. This was reduced to 19.3% for *P. falciparum*-positive samples and clinical malaria prevalence of 8.6% during the next high transmission season. This reduction in the parasite burden in the study population is likely due to the combination of active case detection and ACT of all identified positive cases.

The entomological studies revealed that the study area is under the influence of three vector species *An. fluviatilis* and *An. culicifacies* as the primary vectors and *An. annularis* as the secondary vector for malaria transmission. The climatic conditions in the forest ecotype are more conducive for higher survival of vectors that are associated with a predominance of *P. falciparum* [3]. *An. fluviatilis* is widely distributed in India although its role in malaria transmission varies from place to place depending on the local prevalence of different sibling species [29]. *An. fluviatilis* (species S) is among the most efficient vectors of malaria particularly in hills and foothills of India and it has previously been incriminated as a vector in the tribal districts of Koraput, Orissa [28] and Bastar, Madhya Pradesh [30]. In contrast, other studies have found that *An. fluviatilis* (species T), though prevalent in high densities in the foothills of Shiwalik range was not playing role in malaria transmission [31]. However, *An. fluviatilis* (species T) has been found to be susceptible to *P. vivax* [32] and *P. falciparum* infections (MK Das, unpublished) and has been incriminated as an efficient vector of malaria for transmission in the mountainous areas of the Hormozgan province, south Iran [33]. The role of *An. fluviatilis* (species T) as the primary vector in malaria transmission in this study has been established by way of its high prevalence period coinciding with the peak transmission period, more preference for human biting, high human blood index and susceptibility to plasmodial infections (MK Das, unpublished). The other primary vector, *An. culicifacies* (species C), is responsible for 60–70% of all malaria cases in India despite being predominantly a zoophagic species and being prone to environmental factors with epidemiological implications in different ecotypes [12, 34]. *An. culicifacies* may be regarded as playing a complementary role in maintaining perennial transmission during pre-monsoon and monsoon months where transmission is low. The secondary vector *An. annularis* though primarily zoophagic, exophilic and exophagic, is associated with low human biting rate and sporozoite rate, and has a marginal role in malaria transmission in this study area. While *An. annularis* plays a limited role for malaria transmission in the present study area, it is responsible for year-round transmission as observed in inlands of Odisha [32] and forests of Assam [35]. The presence of the three vector species and their sibling species complexes reported here together with a high prevalence of parasite positive individuals in the study population explains the year-round malaria transmission.

Besides technical inadequacies, the problem posed by malaria in tribal areas of India is multifaceted. Operational constraints such as inaccessible remote areas, poor surveillance, inadequate vector control and general lack of motivation and health awareness all facilitate persistent transmission. Therefore, focus should be on strengthening of health infrastructure at the periphery to ensure early case detection and prompt treatment. This should be supported by quality vector control measures, creating health awareness in the community and ensuring economic growth of the tribal areas. These efforts along with the development of an efficacious malaria vaccine will lead to a sustained reduction in malaria morbidity and mortality.

## Conclusion

In conclusion, the collection of clinical data from a well-characterized tribal cohort from the state of Jharkhand in Eastern India has provided evidence for the presence of NAI against malaria in this hyperendemic region.
